# Correlates of HIV infection and being unaware of HIV status among soon-to-be-released Ukrainian prisoners

**DOI:** 10.7448/IAS.17.1.19005

**Published:** 2014-09-10

**Authors:** Lyuba Azbel, Jeffrey A Wickersham, Yevgeny Grishaev, Sergey Dvoryak, Frederick L Altice

**Affiliations:** 1 Ukrainian Institute on Public Health Policy, Kyiv, Ukraine; 2 Department of Internal Medicine, Section of Infectious Diseases, AIDS Program, Yale University School of Medicine, New Haven, CT, USA; 3 Division of Epidemiology of Microbial Diseases, Yale University School of Public Health, New Haven, CT, USA

**Keywords:** prisoners, substance use, HIV/AIDS, injection drug use, risk behaviours, Ukraine

## Abstract

**Introduction:**

Prisoners bear a disproportionate burden of Ukraine's volatile and transitional HIV epidemic, yet little is known in Eastern Europe about HIV testing, treatment and HIV-related risk among prisoners.

**Methods:**

A nationally representative biobehavioural health survey linked with serological testing was conducted among soon-to-be released prisoners in 13 Ukrainian prisons from June to November 2011.

**Results:**

Among 402 participants, 78 (19.4%) tested HIV seropositive of whom 38 (50.7%) were previously unaware of their HIV status. Independent correlates of HIV infection included drug injection (AOR=4.26; 95% CI: 2.23–8.15), female gender (AOR=2.00; 95% CI: 1.06–3.78), previous incarceration (AOR=1.99; 95% CI: 1.07–3.70) and being from Southern Ukraine (AOR=5.46; 95% CI: 2.21–13.46). Those aware of being HIV-positive reported significantly more pre-incarceration sex- and drug-related HIV risk behaviours than those who were unaware.

**Conclusions:**

Routine rather than risk-based HIV testing and expansion of opioid substitution and antiretroviral therapy among prisoners is urgently needed to reduce HIV transmission in volatile transitional HIV epidemics.

## Introduction

The HIV epidemics in Eastern Europe and Central Asia are among the fastest growing in the world [1]. Together, Ukraine and Russia account for 90% of all newly reported HIV cases in the region [2]. Ukraine is experiencing Europe's worst HIV epidemic [3] with 1.1% of the adult population infected [4]. As in other countries of the former Soviet Union (FSU), Ukraine's HIV epidemic started among people who inject drugs (PWIDs) [4], particularly opioids, [5] with more recent evidence of a transitional generalizing epidemic [6]. HIV prevalence among the estimated 325,000–425,000 PWIDs in Ukraine is estimated to be as high as 41.8% in certain regions [7]. Despite PWIDs accounting for 60.5% of all HIV cases in Ukraine, only 5% of PWIDs are currently receiving antiretroviral therapy (ART), yet over 25% of those not using drugs are on ART [8]. This disproportionate access to ART among PWIDs is indicative of the pervasive trend of stigmatization towards this group, resulting in poor health outcomes [9].

Ukraine has largely relied on criminal justice sanctions to address illicit drug use, favouring incarceration over medical treatment for substance use disorders (SUDs), leading to high concentrations of PWIDs within prisons [10]. Ukraine's incarceration rate (347 per 100,000) is among the world's highest, just after the United States, Russia and Cuba [11]. HIV prevalence in Ukrainian prisons is several-fold higher than in the community, resulting in health disparities among PWIDs. These poor health outcomes are compounded by low ART coverage in prisons, with only 11% of eligible HIV-positive prisoners receiving treatment [12] despite evidence that ART prevents HIV transmission among PWIDs [13, 14]. With documented high levels of drug injection and syringe sharing by HIV-positive prisoners during incarceration [15] and 7000 HIV-positive prisoners released to the community annually [16], Ukraine's criminal justice system (CJS) contributes to poor HIV-related outcomes and perpetuates HIV transmission, making it a volatile, high-risk environment. Though effective alternatives to incarceration exist, in the absence of such policies, Ukraine's CJS remains an important target for public health interventions that address the syndemic of HIV and SUDs among PWIDs by expanding screening, treatment and post-release linkage to care.

In order to better inform HIV testing, treatment and retention strategies within Ukraine's CJS, we examined the independent correlates of HIV infection and differences among those aware and unaware of being HIV-positive among a nationally representative sample of soon-to-be-released prisoners. Such findings may be useful for other countries from Eastern Europe, Central Asia and elsewhere that struggle with transitional HIV epidemics evolving from PWIDs.

## Methods

### Study design

Study design and procedures have been described previously [12]. In brief, a nationally representative cross-sectional health and serosurveillance survey of infectious diseases among 402 soon-to-be-released adult prisoners was conducted between May and November 2011. Eligibility criteria included: 1) ≥18 years of age; 2) <six months remaining in prison sentence; 3) able to provide informed consent; and 4) speaks Ukrainian or Russian. Participants were selected at random from the 87,717 sentenced women and men in medium-security prisons (representing 80.1% of the total prison population), using a stratified sampling strategy [17]. Of the 426 inmates randomly selected, 402 (94.4%) consented and were enrolled. An anonymous identifier linked behavioural and serological results, which was unlinked to personal identifiers unless participants were willing to release their results to prison officials to obtain care. Participants were provided with serological results, post-test counselling and a comprehensive referral packet to community-based medical and social services.

Eligible sites were categorized into one of four geographic regions of the country. Approximately 100 participants were targeted from each of the four regions (north, south, west and east). Each region included two medium-security male-only prisons and one female-only prison. Pre-planned sampling was divided evenly between first time and repeat offenders, and women (20%) were oversampled. Prison facility selection criteria included being large, representative, and non-specialized; HIV/AIDS, addiction, and tuberculosis prison hospitals were not included. HIV testing is available in all prisons through voluntary counselling/testing (VCT) that primarily targets high-risk groups.

### Data collection

Surveys were administered under the supervision of a trained research assistant using computer-assisted survey instruments (CASI) to ensure confidentiality. Nurses performed the phlebotomy. Serological testing was performed using the commercially available One-Step Multi-Infectious Disease Rapid Test Card (InTec Products, Inc. Xiamen, China), including testing for Hepatitis B virus surface antigen (HBsAg), antibody to Hepatitis C virus, ELISA for HIV (1&2) test and a rapid plasma reagin (RPR) test to Treponema pallidum for syphilis (RPR>1:16 was considered positive). Initially reactive HIV serology (sensitivity=99.9%; specificity=100%) was confirmed with the Determine^TM^ HIV-1/2 Western Blot (Abbott Laboratories, Tokyo, Japan). All HIV-seropositive participants underwent reflex CD4 T lymphocyte count assessment using FACS flow cytometry.

### Definitions

All self-reported sex- and drug-risk behaviours were limited to the 30 days prior to arrest in order to minimize recall bias, with the exception of “ever injected drugs,” “ever used drugs” or “ever used opioids,” which refer to lifetime. Alcohol use and tattooing were assessed for the year before incarceration. Poverty was defined as being below the 2011 national Ukrainian poverty line income of <1776 Ukrainian Hryvnia per month (~220 USD). Pre-incarceration drug use refers to opioids, sedatives, amphetamines, hallucinogens, barbiturates or cocaine in the month before incarceration. Multiple-substance use refers to using two or more of the aforementioned substances at least once during that period.

Having moderate to severe depressive symptoms was defined for individuals who scored ≥10 on the 10-item Clinical Epidemiological Scale for Depression (CES-D) [18]; hazardous drinking of alcohol was confirmed if scores were ≥8 for men or ≥5 for women [19] using the Alcohol Use Disorders Inventory Test (AUDIT) [20]; social support was measured using a continuous standardized scale [21]; health-related quality of life (HRQoL) was measured continuously with a physical and mental health summary score using the 36-item short-form (SF-36) Medical Outcomes Survey [22].

### Data analysis

Statistical analyses, using SPSS (version 19.0), involved *t*-test and *χ* ^2^ test for categorical and continuous variables, with significance defined as *p*<0.05. Bivariate and multivariate logistic regression analyses were carried out to determine the correlates of HIV seropositivity. Multivariate logistic regression assessed the independent correlates for HIV-seropositive status if bivariate associations with the dependent variable were significant at *p*<0.05. All variables in the final model were checked for multicollinearity, and tolerance values in the final model were high (>0.90) and all VIF values were low (≤1.10). Injection drug use (IDU) and sex with an HIV-positive partner were included over other sex and drug-risk variables because they are most directly associated with HIV risk and resulted in best goodness-of-fit. Region was included in order to account for region-specific differences. Variables that were kept in the model were tested for interactions with each other. Goodness-of-fit for the final logistic regression was measured using the Akaike Information Criterion (AIC).

### Ethics statement

Institutional Review Boards at the Ukrainian Institute on Public Health Policy and Yale University approved the study. Further safety assurances were provided by the Office for Human Research Protections (OHRP) in accordance with 45 CFR 46.305(c) “Prisoner Research Certification” requirements. Participants provided written informed consent prior to study participation.

## Results

### Description of study participants

Table 1 provides the sample characteristics, including demographics, drug and alcohol use, and pre-incarceration HIV risk behaviours. Overall, 78 prisoners (19.4%) had confirmed HIV infection.

**Table 1:** Selected characteristics of study participants (*N=*402)

Characteristics	*N*=402 (%)
Age	
Mean, years (range)	31.9 (18–58)
≤30 years	212 (52.7)
>30 years	190 (47.3)
Sex	
Male	321 (79.9)
Female	81 (20.1)
High school graduate	
Yes	305 (75.9)
No	97 (24.1)
Ethnicity	
Ukrainian or Russian	381 (95.0)
Other	21 (5.2)
Criminal justice history	
Mean number of lifetime arrests (SD)	5.4 (7.0)
Mean number of previous incarcerations (range)	2.2 (0–11)
Mean current incarceration duration, years (SD)	2.6 (1.9)
Mean time before community release, months (SD)	2.1 (1.7)
In a relationship	
Yes	105 (26.1)
No	297 (73.9)
Below national poverty line	
Yes	243 (60.4)
No	159 (39.6)
Hazardous drinking	
Yes	229 (57.0)
No	167 (41.5)
Substance use 30 days before incarceration	
Any substance use	171 (42.5)
Multiple-substance use	127 (31.6)
Opioid use	138 (34.3)
Amphetamine use	85 (21.1)
Sedatives use	72 (17.9)
Ever injected drugs, lifetime	
Yes	193 (48.7)
No	209 (51.3)
Re-used syringe, container or needle (*N*=144)	
Yes	104 (25.9)
No	40 (10.0)
Major depressive disorder	
Moderate to severe depressive symptoms	158 (40.3)
Social support	
Mean social support score (SD)	2.99 (1.1)
Health-related quality of life	
Mean Physical Composite Score (SD)	47.3 (5.8)
Mean Mental Composite Score (SD)	38.4 (8.9)
HIV seropositive	78 (19.4)
HIV outcomes (*N*=78)	
Mean CD4 count, cells/mL (range)	355.1 (5–1239)
CD4>350	34 (43.6)
CD4≤350	44 (56.4)
Ever prescribed antiretroviral therapy	8 (10.3)
Currently prescribed antiretroviral therapy	5 (6.4)
Hepatitis C antibody	
Negative	161 (40.0)
Positive	241 (60.0)

SD=standard deviation.

**Figure 1:**
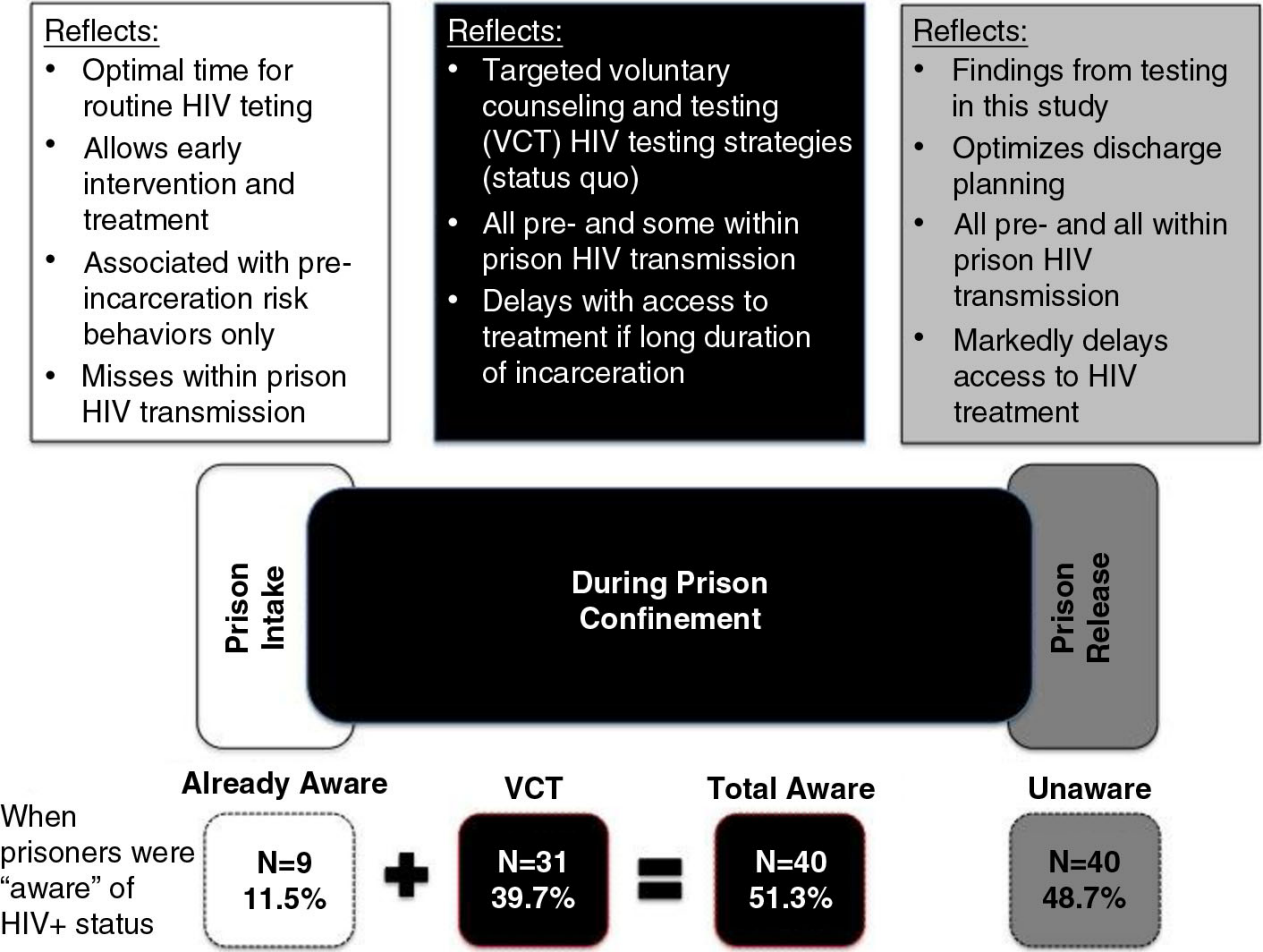
Analytic strategy when prisoners learn about their HIV-seropositive status.

The findings, illustrating when prisoners became aware of their HIV status, are depicted in Figure 1, which provides insights into the attributes of each testing strategy.

**Figure 2:**
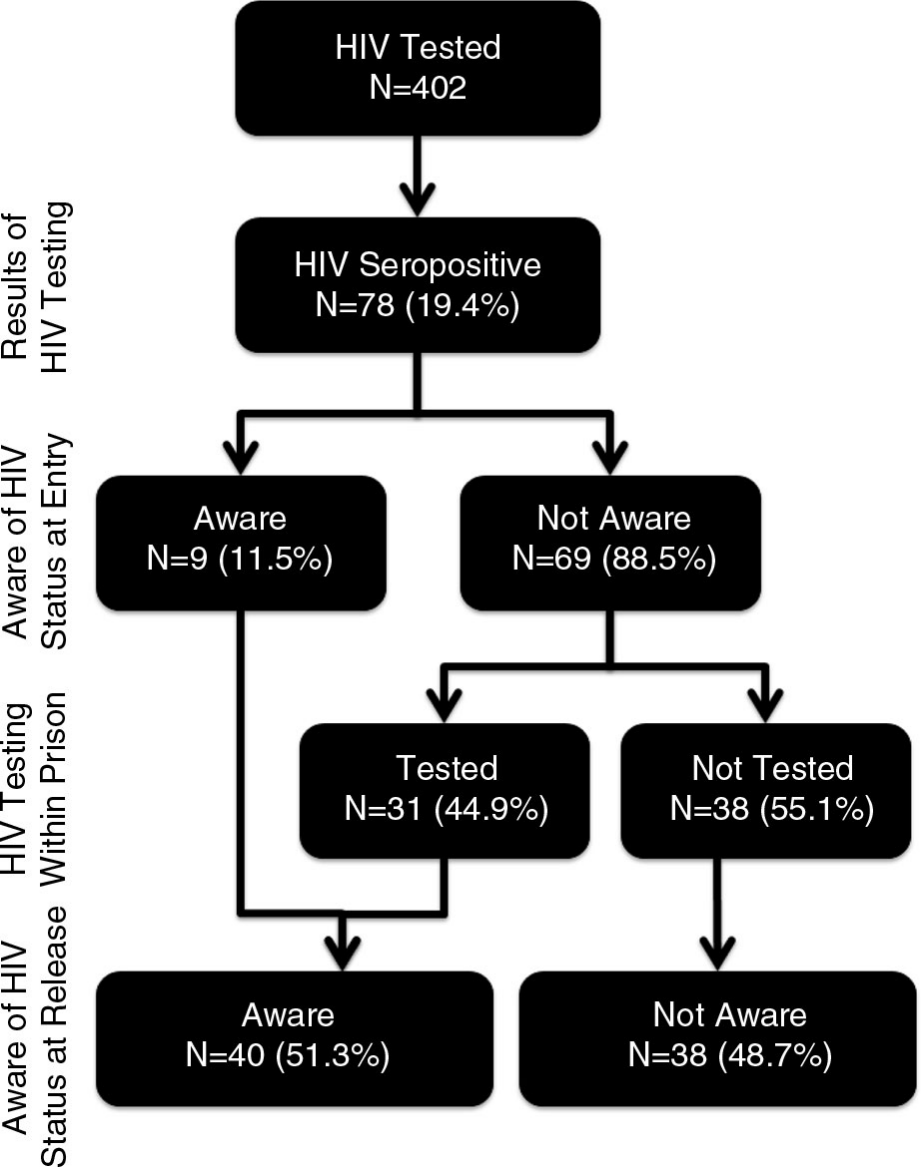
When participants became aware of their HIV status.

Figure 2 depicts knowledge about HIV status, based on pre-specified HIV testing periods (prison entry, during incarceration and at prison release); testing at prison release was testing that occurred as a result of this study. Prison intake reflects a time when “routine” testing is most optimized [23–25]. Nearly all HIV-seropositives were unaware of their status at intake (*N*=69; 88.5%). During imprisonment, 31 of those initially unaware were tested and informed about their status, leaving 38 (48.7%) not knowing their status were it not for this study. Despite 45 HIV-seropositives reporting previous HIV testing, eight of them (17.8%) believed themselves to be HIV-uninfected (of which three had tested negative during the current incarceration) and another eight never received test results. Seven of the eight who had never received their results had undergone testing during their current incarceration.

### Correlates of HIV infection

Correlates of HIV status are presented in Table 2. After controlling for variables significant in the bivariate analysis, multivariable logistic regression analysis showed that having ever injected drugs was the single most important correlate of HIV infection, portending more than a four-fold increased association (AOR: 4.26; 95% CI: 2.23–8.15). Other independent correlates of HIV infection included being female (AOR: 2.00; 95% CI: 1.06–3.78), in Southern Ukraine (AOR: 5.46; 95% CI: 2.21–13.46) and being a recidivist offender (AOR: 1.99; 95% CI: 1.07–3.70).

**Table 2:** Correlates of HIV infection among soon-to-be-released Ukrainian prisoners (*N*=402)

Covariate	Bivariate analysis			Multivariate analysis		

	Unadjusted odds ratio	95% C.I.	*p*	Adjusted odds ratio	95% C.I.	*p*
Female	1.92	1.09–3.37	0.020*	2.00	1.06–3.78	0.032*
Male	Ref.	–	–	Ref.	–	–
Recidivist	2.03	1.22–3.38	0.006*	1.99	1.07–3.70	0.030*
First time offender	Ref.	–	–	Ref.	–	–
Region						
East	Ref.	–	–	Ref.	–	–
West	1.89	0.79–4.55	0.155	1.47	0.56–3.88	0.432
North	3.44	1.52–7.79	0.003*	2.36	1.00–5.67	0.055
South	4.13	1.83–9.32	0.001*	5.46	2.21–13.46	<0.001*
Completed high school	1.57	0.84–2.96	0.158	–	–	–
Income below poverty	1.07	0.64–1.76	0.806	–	–	–
Major depression	1.93	1.16–3.20	0.011*	1.31	0.72–2.39	0.385
Hazardous drinking	0.70	0.42–1.15	0.156	–	–	–
Syphilis	1.04	0.46–2.36	0.920	–	–	–
Told by a doctor they had a sexually transmitted infection	1.95	1.06–3.56	0.031*	1.88	0.97–3.67	0.062
Has partner	1.17	0.67–2.03	0.587	–	–	–
Use in 30 days before incarceration						
Injected drugs	4.07	2.43–6.84	<0.001*	–	–	–
Any substance use	3.45	2.04–5.81	<0.001*	–	–	–
Multiple-substance use	2.87	1.73–4.76	<0.001*	–	–	–
Amphetamine user	1.77	1.01–3.09	0.046*	–	–	–
Opioid user	3.88	2.32–6.49	<0.001*	–	–	–
Sedatives user	2.56	1.45–4.52	0.001*	–	–	–
Lifetime use						
Injected drugs	3.34	1.94–5.73	<0.001*	4.26	2.23–8.15	<0.001*
Any illicit drug	2.52	1.48–4.26	0.001*	–	–	–
Re-used syringe, container or needle	1.52	0.68–3.38	0.307	–	–	–
Sex without condom with at least one person	0.88	0.53–1.47	0.634	–	–	–
Sex without a condom with a HIV+ person	2.91	1.31–6.43	0.008*	1.71	0.72–4.09	0.225
Money or drugs in exchange for sex	0.46	0.06–3.64	0.458	–	–	–
Sex under influence of alcohol	0.33	0.17–0.64	0.001*	–	–	–
Sex under influence of drugs	2.09	1.10–3.96	0.025*	–	–	–
Tattoo from non-professional	0.59	0.30–1.15	0.119	–	–	–
Akaike Information Criterion (AIC)						198.49

* Denotes a significant difference, defined as *p*≤0.05.

### Correlates of being aware of being HIV-positive

Table 3 compares pre-incarceration substance use and HIV risk behaviours among all HIV-seropositive respondents, stratified by whether they knew their HIV-positive status upon entry into prison. Compared to those being unaware, the nine respondents who knew they were HIV-positive upon entry reported significantly higher drug use and sexual risk behaviours in the six months before incarceration; six of these nine “aware” respondents also reported sharing injection equipment, sex without a condom and sex under the influence of drugs (data not shown). Table 3 further explores pre-incarceration risk behaviours among those who were aware and unaware of their status during incarceration (not as a result of this study). Pre-incarceration use of all substances, except sedatives, was significantly more prevalent among those who were aware of their HIV-positive status upon re-entry into the community. Among a number of sexual risk behaviours, having any unprotected sex (*p*=0.031), with an HIV-positive person (*p*=0.048) and under the influence of drugs (*p*=0.016) were each significantly more common before imprisonment among those who were aware of their HIV-seropositive status than among those who were informed only upon prison release. Getting a tattoo from a non-professional did not, however, emerge as significantly different between the groups.

**Table 3:** Pre-incarceration substance use and HIV risk-taking behaviours among HIV-positive prisoners in Ukraine who were aware and unaware of their HIV status upon prison entry and prison release (*N*=78)

Drug-related behaviours	Comparison groups		

	Aware *N*=9 (%)	Unaware *N*=69 (%)	*p*
Prison entry			
Amphetamine use	5 (55.6)	18 (26.1)	0.114
Opioid use	9 (100.0)	38 (55.1)	**0.010**
Sedatives use	5 (55.6)	19 (27.5)	0.124
Substance use	9 (100.0)	43 (62.3)	**0.025**
Multiple-substance use	8 (88.9)	32 (46.4)	**0.029**
Injected drugs	9 (100.0)	46 (66.9)	0.052
Re-used syringe, container or needle	8 (88.9)	30 (43.5)	0.662
Sex-related risks			
Sex without condom with at least one person	9 (100.0)	38 (55.1)	**0.010**
Sex without condom with HIV+ person	4 (44.4)	7 (10.1)	**0.020**
Sex without condom under influence of drugs	8 (88.9)	18 (26.1)	**0.030**
Other risks			
Tattoo from non-professional	0	12 (17.4)	0.340

	**Prison release Aware *N*=40 (%)**	**Unaware *N*=38 (%)**	***p***

Amphetamine use	18 (45.0)	5 (13.2)	**0.003**
Opioid use	29 (72.5)	18 (47.4)	**0.037**
Sedatives use	15 (37.5)	9 (23.7)	0.225
Substance use	33 (82.5)	19 (50.0)	**0.004**
Multiple-substance use	26 (65.0)	14 (36.8)	**0.023**
Injected drugs	33 (82.5)	22 (57.9)	**0.009**
Re-used syringe, container or needle	26 (65.0)	12 (31.6)	**0.001**
Sex-related risks			
Sex without condom with at least one person	28 (70.0)	19 (50.0)	**0.031**
Sex without condom with HIV+ person	9 (22.5)	2 (5.3)	**0.048**
Sex without condom under influence of drugs	20 (50.0)	6 (15.9)	**0.016**
Other risks			
Tattoo from non-professional	6 (15.0)	6 (15.8)	1.000

Bold text signifies significant (*p*<0.05) differences.

**Figure 3:**
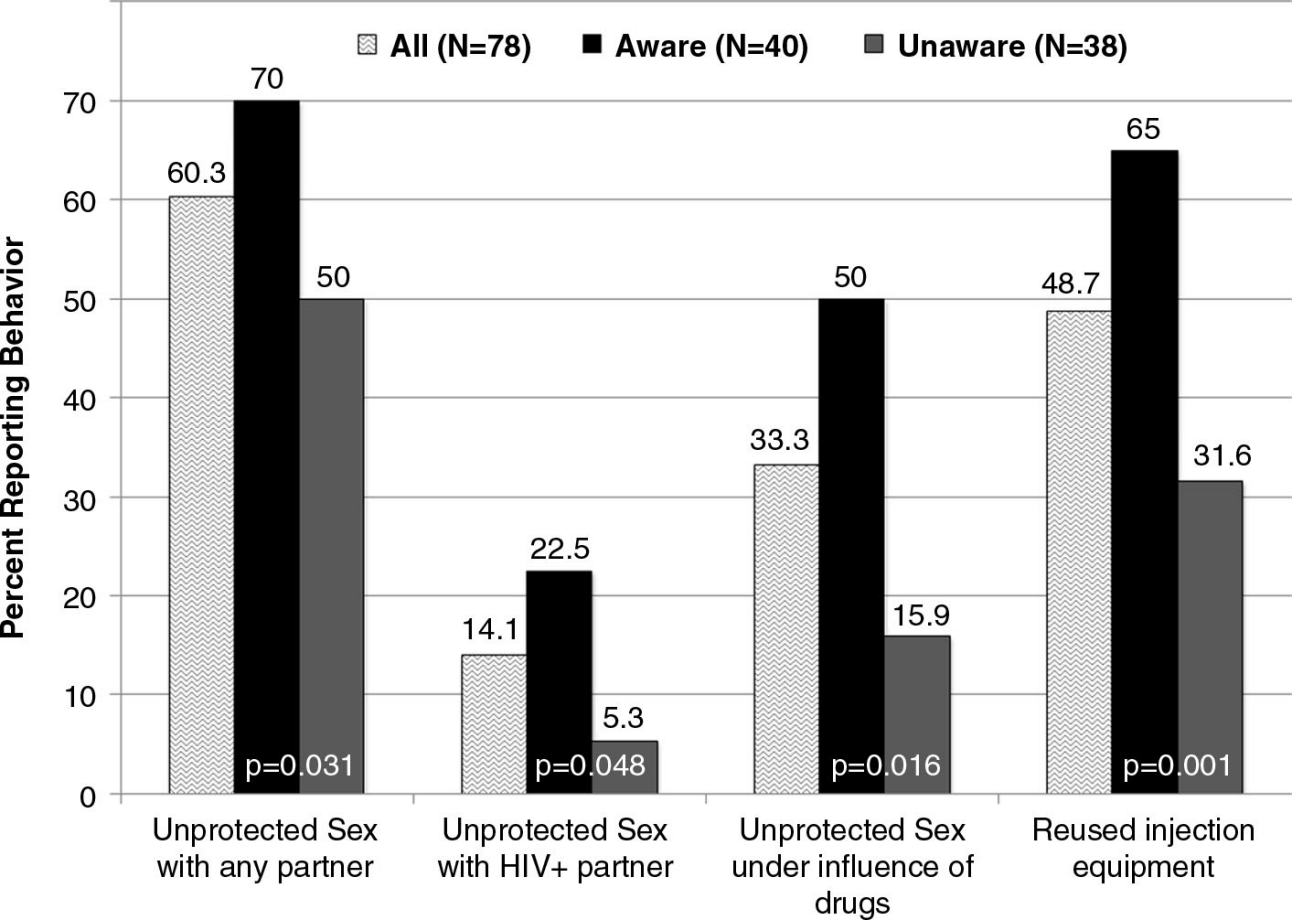
Comparison of pre-incarceration risk behaviours by those who were aware and unaware of their HIV status just prior to prison release (*N*=78).

Figure 3 depicts the proportion of pre-incarceration HIV risk-taking behaviours in all the HIV-seropositive respondents, stratified by who was aware and who was unaware of their status just prior to prison release. Those who were aware reported consistently more risky behaviours.

## Discussion

Though reviews of publicly available data suggest that HIV, drug use and incarceration are inextricably intertwined in this region [26, 27], to our knowledge, this is the first study to directly collect data that examines correlates of HIV infection among prisoners in any country of the FSU, a region with a volatile HIV epidemic concentrated among high-risk groups [29, 30]. This sentinel biobehavioural survey of prisoners confirms this epidemiological impression and provides new insights that inform HIV prevention and treatment strategies. First, HIV prevalence in this representative sample of soon-to-be-released Ukrainian prisoners is substantially higher than recorded in official statistics [28] and several-fold higher (19.4% vs. 1.1%) than in the community [3, 29]. Second, this survey includes only soon-to-be-released prisoners, which provides a window into how effective current voluntary testing strategies are performing. In this case, nearly half of the prisoners would have transitioned to the community remaining unaware of their HIV status. Third, the comparison of those who were aware and unaware of being HIV seropositive near release identified marked differences in risks, pointing to the need to move away from risk-based testing in favour of routine testing strategies.

Meta-analyses and systematic reviews suggest that identification of HIV-seropositive status markedly reduces HIV risk behaviours [30, 31]. Identifying HIV alone, along with self-imposed risk reduction, could markedly contribute to reducing onward HIV transmission after release. Recent data from Ukraine of recently released HIV-positive prisoners suggest that their HIV injection behaviours were lower post-release compared to pre-incarceration behaviours [15]. Though there was evidence that VCT efforts correctly identified 39.7% of HIV-seropositives, a 2009 assessment showed that VCT tested only 1.08% of Ukrainian prisoners [4]. Of the 40 prisoners aware of their status, most (77.5%) were identified during this incarceration. More disturbing, however, is that nearly all (88.5%) participants had been missed by community-based HIV testing strategies. This finding conflicts with national estimates where only 50% of cases, much like we found by the end of imprisonment, were unaware of being HIV seropositive [32]. The increased identification of HIV during imprisonment suggests that in the absence of alternatives to incarceration or improved community-based treatment, prisons can serve as important sites for HIV diagnosis and treatment [33], especially when linked to effective transitional care services [34]. In the absence of diagnosis, people living with HIV/AIDS (PLWHA) often present to care with advanced HIV resulting in increased morbidity and mortality. Early HIV diagnosis improves ART access, which not only benefits individual health but also facilitates secondary prevention [35, 36].

Particularly troubling is the high prevalence of pre-incarceration HIV risk behaviours reported, especially among the HIV-seropositives. Drug use immediately preceding incarceration was independently correlated with being aware of HIV infection. Therefore, using injection risk alone to test patients would have missed a third of all HIV infections. Of the HIV-positive prisoners, those less likely to report risk behavior were significantly less likely to be tested for HIV, indicating that risk-based testing had failed to identify a substantial portion of PLWHA. Not only is HIV testing a hallmark of combined HIV prevention strategies [37, 38], it is also the first essential principle of the Seek, Test and Treat strategy that requires identifying most PLWHA (>90%) to reduce HIV transmission [39]. In many settings, routine HIV testing has been recommended as a cost-effective public health strategy to reduce barriers to testing, including empiric evidence from testing in jails and prisons [23, 24].

Examining drug use, a central driver of HIV transmission among prisoners, provided important insights for future prevention. While any substance use was significantly associated with HIV seropositivity, even when controlling for sexual risks, drug injection independently portended more than a four-fold increased association with HIV and nearly all (87%) of HIV-positive PWIDs injected primarily opioids. For opioid injectors, opioid substitution therapy (OST) with either methadone or buprenorphine remains the single most effective therapy to reduce HIV transmission. Recent data from Ukraine suggest that in the absence of prison-based OST, the prevalence of within-prison drug injection among PLWHA is extraordinarily high and often involves multiple sharing partners [15, 16]. Our data support within-prison HIV transmission since we minimally identify three instances of seroconversion during the current incarceration. Although the actual number of within-prison transmission is potentially much higher since, for 66 of the HIV-positive respondents, we cannot definitively conclude whether they were infected during incarceration or in the community. Even after nearly a decade since OST was introduced in Ukraine [40], it remains absent from the CJS despite plans for pilot study introduction into pre-trial detention settings which have been aborted since political unrest in Ukraine began. Lack of OST within the CJS persists despite the breadth of evidence showing the effectiveness of prison-initiated methadone [41], its benefits for prisoners transitioning to the community [42, 43] and, specifically, evidence that OST reduces HIV transmission risk among PWIDs in Ukraine [44].

Whereas PWID comprise 69% of all PLWHA in Ukraine, they have limited ART access [45]. Central to increasing PWIDs’ access to ART and achieving maximal viral suppression is OST expansion [40, 46, 47], which is recommended by international guidelines [48]. Overcoming stigma faced by PWIDs and reducing structural ART access obstacles is a priority for curbing the epidemic in this high-risk group [49]. Particularly, it is essential to establish effective transitional programmes to community settings to maintain ART benefits [50] which is an evidence-based HIV prevention tool [51]. Of concern here is the fact that half of the HIV-seropositive respondents were unaware of their status, highlighting a critical gap in HIV testing which could be scaled up through routine testing. Our findings suggest high acceptability of routine HIV testing given that very few approached for this study actually refused. Identifying HIV, however, should be linked to care, including ART. Only five patients here received ART; this represents 6.4% of HIV-seropositives, 12.5% of those aware of being HIV seropositive and 22.4% of those meeting ART treatment guidelines (CD4<350).

This study attests to the disproportionate impact HIV has on incarcerated women, portending a two-fold elevated risk. Across Ukraine, evidence suggests that the generalizing HIV epidemic is disproportionately impacting women [2]. Moreover, emerging data from Ukraine suggest that female PWIDs experience increased violence, which restricts access to services, safer sex and injection practices, and, consequently, increases HIV risk [52]. In this study, however, transactional sex was not significantly associated with HIV seropositivity. Even so, women's unique needs often remain unmet by prison services [53], necessitating gender-tailored interventions to address inequalities in care, prevention and rehabilitation services [54, 55].

Breaking the cycle of incarceration in Ukraine is central to HIV prevention efforts [56]. In our sample, having been previously imprisoned increased HIV risk more than two-fold. This underscores the risk posed to the general population, especially considering that an estimated 592 HIV-positive inmates are released to the community each month [29], with about half not knowing their HIV status, suggesting that ongoing sexual risk is contributing to an increasing proportion of new reported HIV cases in Ukraine [4]. Though recent reports document heterosexual transmission's increased contribution in Ukraine's expanding HIV epidemic, data point primarily to transmission to sexual partners of PWIDs [57], causing concern for a generalized epidemic [58].

Nearly all participants became aware of being HIV-positive only after incarceration, thereby limiting exploration of risk behaviours to the pre-incarceration period to ensure inmate safety and confidentiality. Six participants, however, knew their HIV-positive status before incarceration. All of them reported sex without a condom and five had re-used injection equipment and had unprotected sex under the influence of drugs. These data suggest that serosorting may have occurred among sexual partners since knowing one's status markedly reduces risk to others, but further data are needed. Irrespective of whether serosorting occurred or not, the magnitude of risky behaviours, especially under the influence of drugs by those aware of their HIV status remains a concern, and may point to inadequate access to evidence-based HIV risk reduction programmes that effectively curb secondary transmission [59]. At a minimum, recent data point to low coverage of harm reduction programmes such as needle/syringe exchange (NSP) and OST programmes as reasons for increased drug-related risk behaviours [8, 60, 61]. Most studies among HIV-positive PWIDs show reduced sexual risk after learning of one's HIV-seropositive status [62, 63], but this requires further exploration in the current context.

Though these findings provide insights into HIV-related risk and HIV testing strategies for Ukrainian prisoners, this study has limitations. First, the cross-sectional assessment does not determine causality. Second, to protect inmate safety, our study could not directly confirm within-prison HIV risk-taking. Third, recall bias and under-reporting could have occurred, but the use of CASI reduces, although it does not eliminate, reporting bias. Despite these limitations, the findings are a matter of concern and provide insights into future HIV prevention and treatment interventions.

Looking to the future for soon-to-be-released prisoners, the time immediately after release from prison is associated with numerous adverse health consequences [34], including overdose and death [64, 65]. Effective transitional care during this period is crucial since former prisoners return to unstable environments that trigger substance use relapse and increase drug-related death [66]. Optimal transitional care should include linkage to integrated care sites to address medical, psychiatric and addiction needs. Such sites already exist in Ukraine, which document improved health outcomes for HIV-positive PWIDs [67]. Risk reduction programmes are also essential during transition to reduce HIV-risk-taking behaviours [34] and curb HIV transmission to partners of PWID [68] and ultimately to the community [69]. This is especially crucial since low ART coverage among HIV-positive prisoners leaves many without virologic suppression.

## Conclusions

This study is the first to determine the correlates of HIV infection among prisoners in Ukraine, where HIV is concentrated within the epicentre of Europe's worst HIV epidemic. Drug injection, primarily of opioids, is most highly correlated with HIV infection in a representative sample of soon-to-be-released prisoners. Being female and having been previously incarcerated were also associated with HIV infection. Of concern is the fact that half of the HIV-positive prisoners were unaware of their status and this group did not report HIV-related risk behaviours to the same extent as those who were aware. Our findings strongly reinforce the need for five evidence-based practices for prisoners: 1) routine rather than risk-based HIV testing in an expanding and generalized HIV epidemic; 2) OST; 3) expanded prescription of ART to all eligible prisoners; 4) NSPs; and 5) effective transitional care from the prison to the community setting. Though diseases that contribute to health disparities are concentrated within prisons, such as HIV and addiction, good prisoner health is good public health since most prisoners transition to the community and bring with them their medical and social co-morbidity. Coordinated efforts that align health and safety are urgently needed in Ukraine.
